# Human scabies and pediculosis in Ecuador: spatial distribution and environmental determinants

**DOI:** 10.1017/S0031182026102078

**Published:** 2026-05

**Authors:** Andrés Fernando Vinueza-Veloz, Marlon Calispa, Tannia Valeria Carpio-Arias, Estephany Tapia-Veloz, María Auxiliadora Dea-Ayuela, Miguel Mira Naranjo, Pamela Vinueza-Veloz

**Affiliations:** 1 Research Group on Human Food and Nutrition (GIANH), Escuela Superior Politécnica de Chimborazo, Riobamba, Ecuador; 2 Independent Researcher; 3 Departamento de Farmacia, Facultad de Ciencias de la Salud, Universidad Cardenal Herrera-CEU, CEU Universities, Alfara del Patriarca, Spain; 4 Grupo de Investigación en Ciencias Veterinarias, Escuela Superior Politécnica de Chimborazo, Riobamba, Ecuador

**Keywords:** Ecuador, environmental determinants, pediculosis, scabies, spatial distribution

## Abstract

Parasitic diseases, including scabies and pediculosis, pose significant public health concerns, particularly in developing countries. Despite their non-lethal nature, these diseases can cause considerable morbidity. This study aimed to assess the national and subnational burden of scabies and pediculosis in Ecuador during 2021 and explore the spatial correlations between these diseases and environmental factors. An observational, cross-sectional study was conducted using 2021 outpatient data from Ecuador’s Ministry of Public Health. Municipal incidence rates were calculated for scabies (ICD-10 B86) and pediculosis (ICD-10 B85). Local Indicators of Spatial Association analysis was performed to identify epidemiological hot and cold spots. Associations with climatic variables (rainfall, temperature and altitude) were examined using Wilcoxon tests and ordinary least squares regression. A total of 20 722 scabies cases and 3558 pediculosis cases were identified, with national incidences of 118.45 and 20.33 per 100 000 population, respectively. Both diseases were more frequent in women. Scabies hot spots were located in the Coast and Amazon regions and associated with higher rainfall, higher temperature and lower altitude. Pediculosis hot spots were located exclusively in the Amazon region and associated with higher rainfall and higher altitude. Climatic factors explained 24.3% of scabies variance but only 6.3% for pediculosis. This study underscores the importance of climatic and socio-environmental factors in the transmission of scabies and pediculosis and provides valuable epidemiological data for future control efforts in Ecuador.

## Graphical abstract

**Figure:**
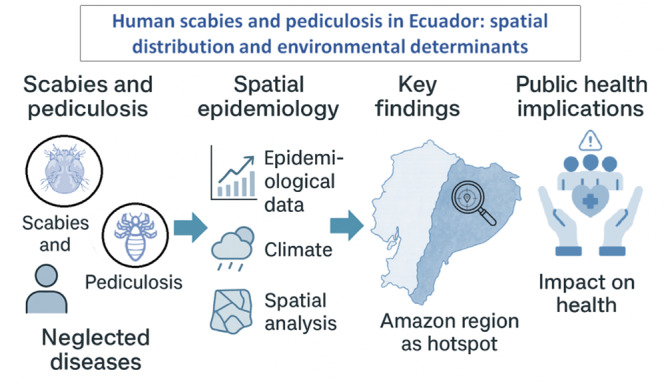

## Introduction

Parasitic diseases remain among the most widespread public health concerns, particularly in developing countries (Antillon et al., 2021). Although they are not typically life-threatening, parasitic diseases caused by arthropods can be deleterious, producing significant morbidity and, in some cases, acting as biological vectors (Robert and Debboun, 2020). Among them, scabies and pediculosis are classified as non-communicable diseases, whose global burden has increased over the last few decades (Murray et al., 2012; Burgess and Silverston, 2015).

Scabies was first described in 1687, when the Italian physician Giovanni Cosimo Bonomo associated the etiological agent with the typical skin injuries (Arlian and Morgan, 2017). In humans, this contagious disease is caused by the mite *Sarcoptes scabiei* var. *hominis*, a small arachnid (∼400 × 300 µm) barely visible to the naked eye. Close skin-to-skin contact is considered the primary route of transmission (Arlian and Morgan, 2017). The parasite feeds on the host epidermis while burrowing into the skin and lays 2–3 eggs per day for up to 6 weeks (Ständer and Ständer, 2021; Sharaf, 2024). After 3 intermedial stages (larva, protonimph and tritonymph), the adult mite emerges onto the skin surface, and following mating, they reinfect the host or spread to another individual. The main clinical manifestation consists of intensely pruritic nocturnal lesions tightly linked to an inflammatory-allergic response to mite-derived products. Secondary bacterial infections with group A *Streptococcus* and *Staphylococcus aureus* are common, potentially leading to local impetigo or even systemic complications (Ständer and Ständer, 2021; Sharaf, 2024).

Importantly, scabies often occurs alongside other parasitic diseases produced by insects such as lice, reflecting the role of socio-environmental conditions on transmission (Feldmeier and Heukelbach, 2009). Among them, human lice *Pediculus humanus capitis* (head louse) and *Pediculus humanus corporis* (body louse) are the primary species responsible for pediculosis (Ko and Elston, 2004). Head pediculosis affects millions of schoolchildren worldwide. Transmission occurs through direct person-to-person contact or indirectly through shared personal items (Tavoletti et al., 2025). Heavily infested children may experience severe itching, leading to sleep disturbances and impaired school performance (Frankowski, 2004). On the other hand, body lice infestations are associated with poor sanitation and are frequently observed among homeless individuals, displaced populations or incarcerated persons living in overcrowded conditions (Heukelbach and Feldmeier, 2004). As in scabies, the characteristic skin lesions associated with pediculosis may lead to a more complex condition known as impetigo (Ko and Elston, 2004). Beyond the classical view as a skin disease, the parasites can also be associated with other disorders and illnesses. For instance, head and body lice can contribute to severe anaemia and may also act as vectors for the spread of typhus, relapsing fever or bartonellosis (Angelakis and Raoult, 2014; Woodruff and Chang, 2019; Amanzougaghene et al., 2020).

The resurgence and periodic outbreaks of ectoparasitic infestations have been traditionally difficult to explain, and many factors seem to contribute to their spread (Laganà et al., 2024). One important determinant is the parasite’s capacity to survive in the external environment while maintaining infectivity (Korycinska et al., 2020). For example, low temperatures combined with high relative humidity have been associated with increased survival of scabies mites (Liu et al., 2016).

In Ecuador, the epidemiology of pediculosis and scabies remains poorly characterized, limiting the efficient allocation of scarce public health resources for their control and prevention. Therefore, this study aims to estimate the national and local burden of scabies and pediculosis in Ecuador and to evaluate the spatial correlations between both diseases with climatic conditions in 2021.

## Materials and methods

This is an observational, cross-sectional study based on available data from 2021, conducted in Ecuador across 3 different regions of the country: the Coast, Sierra and Amazon region, at the municipal level (221 municipalities). The Coastal region is located on the western lowlands along the Pacific Ocean. The Sierra region lies in the highlands of the Andes mountain range, while the Amazon region is located on the eastern lowlands, forming part of the Amazon rainforest.

### Data sources

Cases of scabies and pediculosis were extracted from the database of the Ministerio de Salud Pública (MSP) of Ecuador entitled ‘Consulta Externa 2021’ (free access database, available at the following link: https://datosabiertos.gob.ec/dataset/?organization=ministerio-de-salud-publica). This database compiled the diagnoses obtained (under the ICD-10 codification) from patients attending outpatient consultations in the public network of the national health system. This network covers 80% of the Ecuadorian population, and its health centres are distributed in urban and rural areas throughout the country (Lucio et al., 2011). The case definition of pediculosis (*Pediculosis corporis* and *Pediculosis capitis*) was established by ICD-10 codes: B850 (pediculosis due to *Pediculus humanus capitis*), B851 (pediculosis due to *Pediculus humanus corporis*) and B852 (pediculosis not otherwise specified). The case definition of scabies was established by the ICD-10 code: B86 (scabies). Only newly diagnosed cases were included. Demographic information on cases was age (years), sex (male and female), ethnic self-identification (mestizo, white, indigenous and black) and municipality of usual residence.

### Data analysis

The national incidence was obtained by dividing the number of cases of each disease by the national population projection for the year 2021 (16 938 986 persons). Municipal incidences were obtained by aggregating the cases in each municipality of usual residence and dividing this figure by the projected municipal population for the year 2021. National incidences use rates per 100 000 inhabitants, while municipal incidences use rates per 1000 inhabitants. Demographic characterization of pediculosis and scabies cases was analysed, stratified by sex. The Chi-square test was applied to evaluate differences in categorical variables (ethnicity and region), while the Wilcoxon test was used to assess age in relation to sex. Age did not show a parametric distribution (Shapiro–Wilk: *P* > 0.001).

### Environmental variables

Three environmental variables were evaluated at the municipal level: annual rainfall (mm/year – RAIN), annual mean temperature (°C – TEMPERATURE) and mean altitude (metres above sea level – ALTITUDE). RAIN and TEMPERATURE correspond to the average values between 1981 and 2021, as provided by CHELSA. The variable ALTITUDE represents the mean altitude of each municipality derived from a digital elevation model, at 30 m spatial resolution (Karger et al., 2020; Brun et al., 2022). Climate data were derived from the CHELSA V2.1 model (Climatologies at high resolution for the earth’s land surface areas, available at: https://chelsa-climate.org). CHELSA is a 1 km-scale global climate dataset designed to provide free access to high-resolution climate data for research purposes. This model is based on a mechanistic statistical disaggregation of global reanalysis data or global climate circulation model output (Karger et al., 2020; Brun et al., 2022). To examine the relationship among these environmental predictors, Pearson correlation coefficients were calculated. Municipality boundaries (polygons) were obtained from the Instituto Geográfico Militar del Ecuador (QGIS-Development-Team, 2021).

### Spatial autocorrelation analysis

Prior to the spatial correlation analyses, a matrix of spatial weights was structured using inter-municipal queen continuity, maintaining an order of first-degree contiguity and excluding the Galapagos Islands municipalities. Spatial autocorrelation was assessed using the Moran Global Index. Local Indicators of Spatial Association (LISA) analysis was used to identify spatial patterns in the incidence of both diseases. The result identified spatial *clusters* whose spatial location is not random (Anselin et al., 2009). Municipal *clusters* with high disease incidence that were surrounded by municipalities with high disease incidence were identified as epidemiological hot spots (*high-high clusters* in the graph). Municipal *clusters* with low disease incidence that were surrounded by municipalities with also low incidence were identified as epidemiological cold spots (*low-low clusters* in the graph). The global and local autocorrelation results were randomized for 999 simulations. To evaluate whether climatic conditions differed between epidemiological hot and cold spots, median values of RAIN, TEMPERATURE and ALTITUDE were compared using the Wilcoxon test.

### Spatial regression analysis

To quantify the association between environmental covariates and disease incidence across all municipalities, we fitted ordinary least squares (OLS) regression models. Due to the strong collinearity between temperature and altitude (*r* = −0.992), altitude was excluded to avoid variance inflation. Temperature and annual precipitation were entered as independent variables. Separate models were fitted for scabies and pediculosis incidence. Residuals from each OLS model were tested for spatial autocorrelation using Moran Global Index, applying the same queen contiguity spatial weights matrix. Significant residual autocorrelation indicates that unmeasured spatially structured factors remain after accounting for climatic variables.

All spatial analyses were carried out in RStudio version 2023 (packages: janitor, dplyr, ggstatsplot and spdep), GeoDa version 1.22 and QGIS version 3.36 (Anselin et al., 2009; QGIS-Development-Team, 2021). A significance level of *α* = 0.05 was used for all statistical tests.

## Results

### Demographic characterization of pediculosis and scabies cases

During the year 2021, 17 493 594 outpatient medical consultations were attended in the MSP. A total of 3558 cases of pediculosis were identified, 98.21% (*n* = 3 494) due to *P. humanus capitis* and 1.79% (*n* = 64) due to *P. humanus corporis*. In addition, a total of 20 722 cases of scabies were identified. The national incidence per 100 000 population was 20.33 and 118.45 for pediculosis and scabies, respectively.

Both diseases were more frequent in women than in men (scabies: male = 44.6% and female = 55.4%; pediculosis: male = 19% and female = 81%). With respect to age, cases of pediculosis were more frequent in younger people than in cases of scabies. The ethnic group with the highest incidence of both diseases was mestizo, followed by indigenous. No different ethnic composition was identified in relation to sex in both diseases (*P* > 0.05). Most cases of pediculosis were reported in the Amazon region, while most cases of scabies were reported on the Coastal (Table 1).

**Table 1.** Demographic characterization of scabies and pediculosis cases in Ecuador during the year 2021 Table 1 long description.

	Pediculosis				Scabies			
Variable	*N* = 3558	*M* = 678	*F* = 2880	*P*	*N* = 20 722	*M* = 9256	*F* = 11 466	*P*
**Age (median ± IQR)**	14.12 (15)	12.35 (16)	14.54 (15)	<0.001	19.37 (21)	15.72 (19)	22.32 (21)	<0.001
**Ethnicity**				0.2				0.7
White	6 (0.2%)	2 (0.3%)	4 (0.2%)		69 (0.4%)	32 (0.4%)	37 (0.4%)	
Indigenous	1405 (43%)	289 (46%)	1116 (43%)		2600 (15%)	1142 (15%)	1458 (14%)	
Mestizo	1813 (56%)	330 (53%)	1483 (57%)		14 633 (82%)	6369 (82%)	8264 (82%)	
Black	23 (0.7%)	5 (0.8%)	18 (0.7%)		592 (3.3%)	271 (3.5%)	321 (3.2%)	
NA	311	52	259		2828	1442	1386	
**Region**				0.004				0.001
Amazon	1602 (45%)	307 (46%)	1295 (45%)		4661 (23%)	2127 (23%)	2534 (22%)	
Coast	789 (22%)	120 (18%)	669 (23%)		11 204 (55%)	5044 (55%)	6160 (54%)	
Highlands	1132 (32%)	240 (36%)	892 (31%)		4544 (22%)	1919 (21%)	2625 (23%)	
NA	35	11	24		313	166	147	

*N*, total cases; M, male sex; F, female sex; NA/ethnicity, the case was not identified with any of the above ethnicities; NA/region, not assigned to the studied regions; IQR, interquartile rank.

### Spatial distribution of scabies and pediculosis incidence

**Figure 1.**
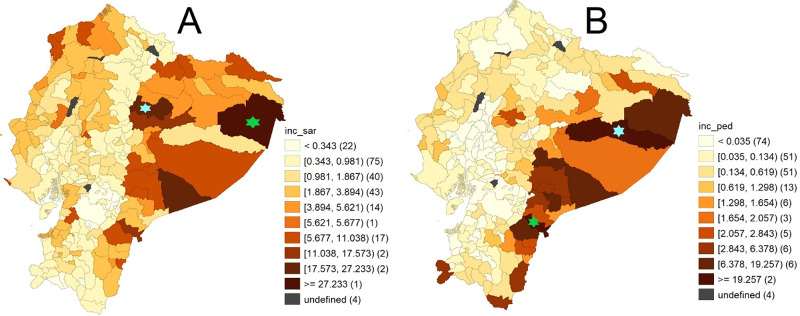
Choropleth maps of municipal incidence of scabies (A) and pediculosis (B) in Ecuador during the year 2021. Each map uses a colour scale, where each colour represents a natural range of disease incidence per 1000 inhabitants (incidence represented in brackets). In brackets the number of municipalities belonging to each interval, polygons defined as ‘undefined’ are areas with disputed territorial boundaries with neighbouring municipalities. For each disease, the green star indicates the location of the municipality with the highest incidence, the light blue star indicates the location of the second municipality with the highest incidence. Figure made with GeoDa version 1.22. Figure 1 long description.

Overall, the Amazon region had a higher incidence of both pediculosis (1.57) and scabies (4.78) compared to the Coast (pediculosis: 0.04 and scabies: 1.57) and Highlands (pediculosis: 0.07 and scabies: 0.65; *P* < 0.001; Figure 1). The post hoc analysis for scabies revealed significant differences between the Amazon, Coast and Highlands regions (*P* < 0.001). However, no significant differences were identified between the Coast and Highlands regions (*P* > 0.05).

Within the Amazon region, the municipalities most affected by scabies were Aguarico (27.23 cases per 1000 population) and Archidona (23.06 cases per 1000 population; see Figure 1A). The highest incidence of pediculosis was observed in San Juan Bosco (22.77 cases per 1000 population) and Arajuno (19.25 cases per 1000 population), also located in the Amazon region (see Figure 1B). In contrast, municipalities in the Sierra region recorded a generally low incidence of both conditions. Municipal-level incidences of scabies and pediculosis in Ecuador in 2021 are detailed in the Supporting Information (Table S1).

### Identification of epidemiological hot and cold spots for scabies and pediculosis

The Moran Global Index value for the municipal incidence of scabies was 0.25 (*Z* = 6.18, Pseudo *P* = 0.001), while for pediculosis it was 0.331 (*Z* = 8.06, Pseudo *P* = 0.002), indicating that the incidence of both diseases is spatially autocorrelated.

**Figure 2.**
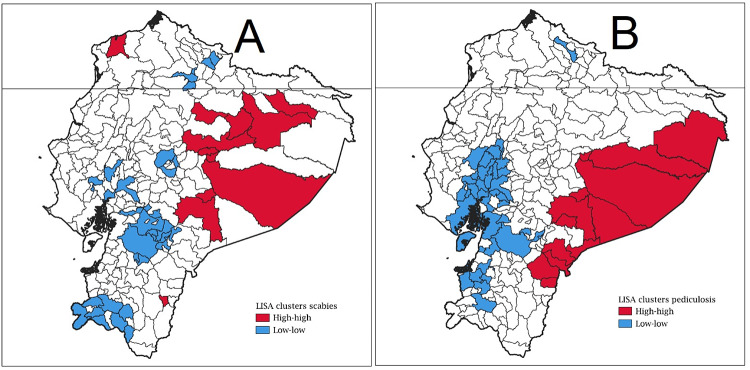
LISA analysis: Identification of epidemiological hot and cold spots of scabies (A) and pediculosis (B) in Ecuador during the year 2021. Hot spots are shown in red and cold spots in blue. The municipalities that were not part of the epidemiological hot or cold spots are in white. Figure made with QGIS version 3.36. Figure 2 long description.

The LISA analysis identified 12 epidemiological hotspots for scabies in the Coast (province of Esmeraldas) and the Amazon regions (provinces of Sucumbíos, Pastaza, Napo, Orellana, Morona Santiago and Zamora Chinchipe). Additionally, 36 epidemiological cold spots for scabies were detected in the Coast region (provinces of Guayas and Los Ríos) and across all provinces in the Highlands region, except for Bolívar province (see Figure 2A).

For the municipal incidence of pediculosis, the LISA analysis identified 13 epidemiological hotspots in the Amazon region (provinces of Pastaza, Orellana, Morona Santiago and Zamora Chinchipe). Additionally, 36 epidemiological cold spots were in the Coast region (provinces of Guayas, El Oro and Los Ríos) and the Highlands (provinces of Azuay, Carchi, Cañar and Loja; see Figure 2B).

### Study of the climatic determinants of the municipal incidence of scabies and pediculosis

**Figure 3.**
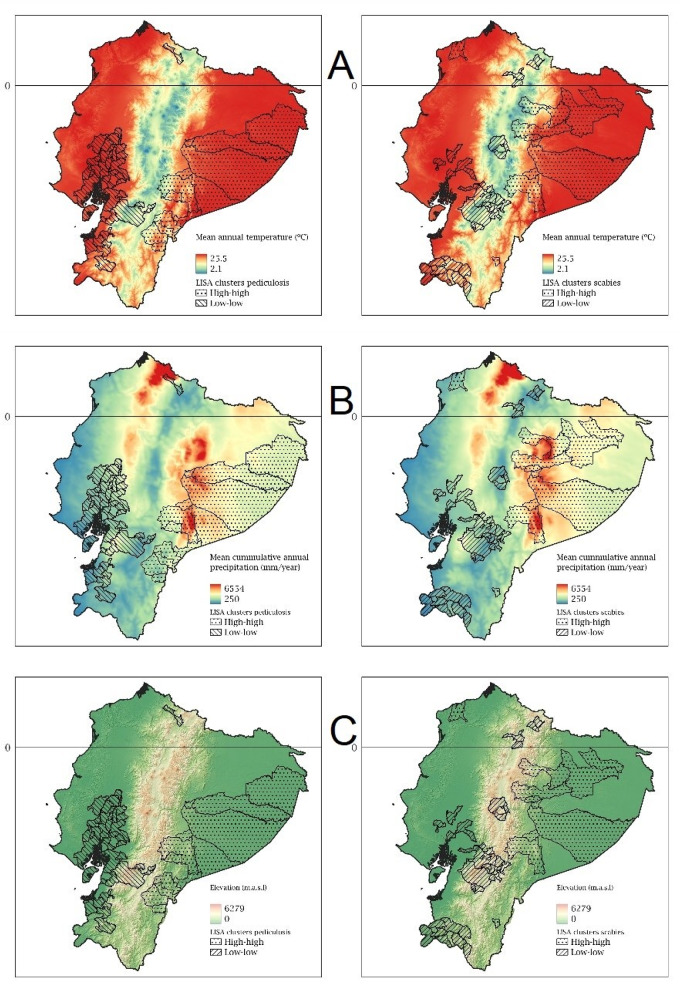
Temperature (A), precipitation (B) and elevation (C); and their relationship with epidemiological hot and cold spots for scabies (right column) and pediculosis (left column). Figure 3 long description.

At the national level, the recorded temperature ranged from 7.68 °C to 25.2 °C, with a median of 21 °C and an interquartile range of 10.6 °C. Annual rainfall (RAIN) ranged from 413 mm to 4784 mm, with a median of 1620 mm and an interquartile range of 1229 mm (see Figure 3). Altitude ranged from 3.33 masl to 6310 masl, with a median of 1035 masl and an interquartile range of 2318 masl. Correlation analysis revealed a strong negative linear relationship between temperature and altitude (*r* = −0.992). In contrast, precipitation showed negligible correlation with both temperature (*r* = 0.04) and altitude (*r* = −0.03).

The comparative analysis between hot and cold epidemiological points and their relationship with climatic variables can be seen in Table 2 and Figure 3. Regarding scabies, this was related to higher RAIN, TEMPERATURE and lower ALTITUDE compared to cold epidemiological points. In the case of pediculosis, hot epidemiological sites had higher RAIN and ALTITUDE compared to cold epidemiological sites (see Table 2 and Figure 3). The results, therefore, suggest that the climatic variables evaluated are different between hot and cold epidemiological hot spots for both diseases.

**Table 2.** Climatic differences between epidemiological hot and cold spots for pediculosis and scabies in Ecuador during the year 2021 Table 2 long description.

	Rain (mm/year)			Temperature (°C)			Altitude (masl)		
	Hot spot	Cold spot	*P*	Hot spot	Cold spot	*P*	Hot spot	Cold spot	*P*
**Scabies**	3752.57	1296.12	<0.001	21.72	12.88	0.02	929.93	2613.97	0.03
**Pediculosis**	2929.19	1567.97		21.15	23.9	0.18	1 032.55	58.84	<0.01

mm, millimetres; °C, degree Celsius.

### Spatial regression analysis

The OLS model for scabies explained 24.3% of the variance in municipal incidence (*R*^2^ = 0.243, *F*(2, 215) = 34.54, *P* < 0.001). Both temperature and precipitation were significant positive predictors (Table 3). For pediculosis, the model explained only 6.3% of the variance (*R*^2^ = 0.063, *F*(2, 215) = 7.29, *P* = 0.001). Precipitation was a significant positive predictor, whereas temperature showed no association (Table 3).

**Table 3.** Ordinary least squares regression results for scabies and pediculosis incidence in Ecuador during the year 2021 Table 3 long description.

Variable	Scabies *β* (SE)	*P*	Pediculosis *β* (SE)	*P*
**Intercept**	−2.661 (0.763)	<0.001	0.265 (0.650)	0.684
**TEMPERATURE (°C)**	0.115 (0.035)	0.001	0.014 (0.036)	0.643
**RAIN (mm/year)**	0.0014 (0.0002)	<0.001	0.0006 (0.0002)	<0.001
***R*^2^**	0.243		0.063	
**Adjusted *R*^2^**	0.236		0.055	
***F* (*df* = 2.215)**	34.54	<0.001	7.29	0.001

*β*, unstandardized coefficient; SE, standard error; *df*, degrees of freedom. Dependent variable: municipal incidence per 1000 population. Independent variables: TEMPERATURE (°C) and RAIN (mm/year). Models were fitted separately for each disease. Ordinary least squares model assumptions were checked (normality of residuals and homoscedasticity), and no serious violations were found.

Residuals from the scabies model exhibited modest but significant spatial autocorrelation (Moran’s *I* = 0.117, *P* = 0.001), while residuals from the pediculosis model showed stronger spatial autocorrelation (Moran’s *I* = 0.283, *P* < 0.001). These findings indicate that climatic factors alone do not fully account for the spatial distribution of either disease, and that unmeasured spatially structured covariates – likely socioeconomic, behavioural or healthcare–related – play an important role.

## Discussion

Scabies and pediculosis represent an important source of morbidity worldwide (Cox et al., 2021). Historically, both diseases have been reported more frequently in Asia and Latin America than in Europe (Karimkhani et al., 2017; Hatam-Nahavandi et al., 2020). However, these studies also highlight the scarcity of epidemiological data from the Andean region (Ecuador, Peru and Bolivia). The present study aimed to characterize the incidence of pediculosis and scabies in Ecuador during the year 2021, as well as the climatic variables that could be associated with their incidence.

According to the Global Burden of Disease (GBD) study, scabies affected an estimated 204.15 million people worldwide in 2015 (Karimkhani et al., 2017). In this analysis, the Andean region had an incidence of 70 cases per 100 000 inhabitants in 2015, which is lower than the incidence identified in our study (Karimkhani et al., 2017). This discrepancy may reflect the limited availability of epidemiological data from North and Latin America included in the GBD estimations (Cox et al., 2021), potentially leading to an underestimation of the regional burden.

Although diseases such as pediculosis or scabies have low mortality rates, their greatest impact arises from the morbidity associated with scratching and secondary bacterial superinfection (Karimkhani et al., 2017; Hatam-Nahavandi et al., 2020). Scabies alone is estimated to account for 71.11 disability-adjusted life years per 100 000 population (Karimkhani et al., 2017).

Pediculosis, in contrast, has received comparatively less global attention, and comprehensive estimates of its prevalence, morbidity or mortality are lacking. However, due to its infection patterns, the school-aged population has been relatively well studied, with approximately 19% of schoolchildren infected worldwide (Hatam-Nahavandi et al., 2020). In Latin American and Caribbean countries, this figure may reach up to 33% (Hatam-Nahavandi et al., 2020), highlighting significant regional heterogeneity.

### Characterization of scabies and pediculosis cases

Consistent with previous reports (Karimkhani et al., 2017; Hatam-Nahavandi et al., 2020), our findings indicate a higher frequency of both pediculosis and scabies in females (see Table 1). Among children, pediculosis has been reported to be up to 2.7 times more frequent in girls (prevalence: 19%) than in boys (prevalence: 7%; Hatam-Nahavandi et al., 2020). Several factors may explain this difference. Hair length has been identified as a potential contributor, as longer hair can facilitate lice dispersal and increase the efficiency of host-to-host transmission (Heukelbach et al., 2005; Lesshafft et al., 2013). In addition, mechanical removal of nits using fine-toothed combs may be more challenging in longer hair, potentially leading to persistent infestations (Heukelbach et al., 2005; Lesshafft et al., 2013). Behavioural factors may also influence transmission dynamics. During childhood, girls may engage more frequently in indoor activities involving closer interpersonal contact, whereas boys are more likely to participate in outdoor play, which may reduce prolonged head-to-head contact (Heukelbach et al., 2005; Lesshafft et al., 2013). In adults, women often have closer head-to-head contact with children, which may further increase exposure risk (Heukelbach et al., 2005; Lesshafft et al., 2013).

Regarding scabies, prolonged close skin-to-skin contact remains the principal route of transmission. The International Alliance for the Control of Scabies (IACS) defines relevant exposure scenarios as sharing a bed, sleeping in the same room, occupational exposure or participation in sports involving sustained physical contact (Engelman et al., 2020). The increased physical interaction among women, influenced by social habits and child-rearing behaviours, has been identified as a risk factor for scabies (Engelman et al., 2020), which may also explain our findings. However, diagnostic bias should also be considered, as women may seek medical care more frequently than men, potentially inflating recorded incidence (Engelman et al., 2020).

With respect to ethnicity, Indigenous individuals accounted for 43% of pediculosis cases, while mestizo individuals represented 56%. However, less than 10% of the total population in Ecuador self-identifies as Indigenous (INEC, 2010). This finding suggests that pediculosis is frequent in both cases, independently of ethnicity.

### Spatial distribution and association with climatic determinants

Scabies hot spots were associated with warmer, wetter and lower-altitude regions compared with cold spots (Table 2). In particular, the mite *Sarcoptes scabiei* var. *hominis* has a monoxenous life cycle, characterized by complete dependence on the human host and limited resistance to environmental stressors, such as dehydration, once detached from the skin host (Heukelbach and Feldmeier, 2006). Nevertheless, its survival outside the host can extend up to 68 h under humid conditions typical of tropical climates (75% relative humidity), thereby increasing the likelihood of transmission through prolonged viability of infective stages (Hay et al., 2012; Laganà et al., 2024).

Similar patterns have been reported in other regions. In China, scabies incidence was positively associated with humidity (*r* = 0.19, *P* < 0.01) but inversely correlated with temperature (*r* = −0.15, *P* < 0.01; Liu et al., 2016). Likewise, in Israel, a 1.3-fold higher incidence of scabies was identified during cold and wet months compared to warm and dry months (Mimouni et al., 2003). Our findings align with these studies regarding the role of humidity but differ with respect to temperature. This suggests that neither temperature nor humidity alone fully determines transmission dynamics; rather, a specific combination of climatic conditions appears necessary to sustain parasite survival and spread. Importantly, scabies transmission cannot be explained solely by climatic variables. Structural social determinants associated with poverty, including overcrowding, limited education and inadequate sanitation, play a critical role (Vinueza Veloz et al., 2024). Overcrowding, for instance, increases the infectivity of *Sarcoptes scabiei* var. *hominis* due to the close physical contact it facilitates (IACS 2020) (Engelman et al., 2020). In Ecuador, the Amazon region has the highest rate of overcrowding (17.2%) compared to the Coast (10%) and the Highlands (5%) (Pillalaza-Piguave, 2022).

Regarding pediculosis, our study concludes that its incidence is higher in wetter regions and at higher altitudes compared to cold epidemiological hotspots (Table 2). Factors such as altitude, annual temperature range, slope and mean diurnal range have been identified as key climatic and environmental determinants influencing the occurrence of pediculosis in other parts of the world, such as Iran (Adham et al., 2020). Interestingly, the same study indicates that when altitude exceeds 1500 masl, the prevalence of pediculosis decreases. Our data support this epidemiological pattern, as localized pediculosis hotspots were observed at altitudes of up to 1032.55 masl, suggesting that altitude plays a significant role in shaping the distribution of both the parasite and the disease.

Complementing the hot spot analysis, our spatial regression models quantified the contribution of climatic factors across all municipalities. Temperature and precipitation explained 24% of scabies variance, confirming that climate contributes to its distribution but is far from the whole explanation. The significant residual spatial autocorrelation (Moran Global Index = 0.12, *P* = 0.001) points to unmeasured spatially structured factors – such as overcrowding, which is highest in the Amazon (17.2%) and closely aligns with scabies hot spots. For pediculosis, climate explained only 6% of variance, and residual autocorrelation was much stronger (Moran Global Index = 0.28, *P* < 0.001). This indicates that non–climatic factors – school contact networks, hygiene practices and health education – dominate its spatial pattern. These regression findings do not replace but rather strengthen the hot spot analysis: they confirm that while climate helps locate high–risk areas for scabies, both diseases are primarily driven by socioeconomic and behavioural determinants, which should guide intervention strategies.

### Methodological strengths and weaknesses of the study

This study is the first in Ecuador and the Andean region to address pediculosis and scabies using both national and subnational perspectives. It also contributes valuable data for future GBD estimates for these 2 conditions. The multidisciplinary approach enabled the identification of high- and low-incidence areas, as well as the analysis of climatic factors influencing the epidemiological patterns of these diseases. Although information on the socioeconomic and cultural factors associated with these diseases is limited, our findings can help guide health initiatives focused on the primary prevention of pediculosis and scabies.

The present study has several limitations. First, the reported incidence likely underestimates true disease occurrence, as our analysis relied exclusively on public healthcare records, excluding private consultations and unreported cases not reaching any facility. Second, the cross-sectional design using only 2021 data precluded investigation of temporal dynamics, such as seasonal transmission patterns, year-to-year variations or stability of spatial clusters over time. Future longitudinal studies incorporating multi-year surveillance data are essential to understand temporal trends and determine whether identified hot spots persist or shift in response to climatic or socioeconomic changes.

## Supporting information

10.1017/S0031182026102078.sm001 Vinueza-Veloz et al. supplementary material Vinueza-Veloz et al. supplementary material

## Supplementary material

The supplementary material for this paper can be found at https://doi.org/10.1017/S0031182026102078.

## Figure 1 Long description

A) The map of Ecuador displays municipal incidence of scabies per 1000 inhabitants in 2021. The legend ranges from less than or equal to 0.343 (23 municipalities) to 27.53 to 73.73 (2 municipalities). The highest incidence is marked by a green star and the second highest by a light blue star. Undefined areas are shown with disputed boundaries. B) The map shows municipal incidence of pediculosis per 1000 inhabitants in 2021. The legend ranges from less than or equal to 0.005 (74 municipalities) to 9.27 to 23.67 (2 municipalities). The highest incidence is marked by a green star and the second highest by a light blue star. Undefined areas are similarly marked. Both maps indicate higher incidence in the Amazon region compared to the Coast and Highlands.

Navigate back to Figure 1.

## Figure 2 Long description

A) The map of Ecuador displays LISA clusters for scabies. Hot spots are marked in red, primarily located in the Coast and Amazon regions. Cold spots are shown in blue, concentrated in the Coast region and across the Highlands, excluding Bolívar province. B) The map illustrates LISA clusters for pediculosis. Red areas indicate hot spots, covering large portions of the country, while blue areas represent cold spots, scattered in various regions. Municipalities not part of hot or cold spots are in white. Both maps highlight spatial patterns of epidemiological significance.

Navigate back to Figure 2.

## Figure 3 Long description

A) Two maps display mean annual temperature in degrees Celsius, with a gradient from lower to higher temperatures. The left map highlights pediculosis and the right map focuses on scabies. Hatched regions indicate hot spots and dotted areas show cold spots. B) Two maps illustrate mean cumulative annual precipitation in millimeters. The left map shows pediculosis and the right map shows scabies. Hatched and dotted areas represent hot and cold spots, respectively. C) Two maps depict elevation in meters above sea level. The left map is for pediculosis and the right map is for scabies. Hatched regions indicate hot spots and dotted areas show cold spots. Each map uses a gradient to represent the variable, with specific areas marked for epidemiological significance. The maps are oriented with north at the top and internal boundaries are visible but not labeled. The legend provides specific ranges for temperature, precipitation and elevation, enhancing interpretability. Hot spots tend to cluster in warmer, wetter and lower areas, while cold spots are in cooler, drier and higher regions.

Navigate back to Figure 3.

## Table 1 Long description

The table summarizes 2021 Ecuador cases of pediculosis and scabies by sex, reporting median age with interquartile range plus distributions by ethnicity and region, with p values for sex differences. Pediculosis includes 3,558 cases, mostly female (2,880 vs 678 male), with a median age about 14 years; males are younger than females and the age difference by sex is statistically significant. Scabies includes 20,722 cases, with more females than males (11,466 vs 9,256), and a higher median age about 19 years; males are younger than females and the age difference by sex is statistically significant. Ethnicity is dominated by Mestizo for both conditions (about 56 percent for pediculosis and about 82 percent for scabies); Indigenous is much higher in pediculosis (about 43 percent) than in scabies (about 15 percent), and ethnicity does not differ significantly by sex for either condition. Region differs by sex for both conditions: pediculosis is concentrated in the Amazon (about 45 percent) with fewer cases on the Coast (about 22 percent) and Highlands (about 32 percent), while scabies is concentrated on the Coast (about 55 percent) with smaller shares in the Amazon and Highlands (each about 22 to 23 percent). Some cases are listed as not assigned for ethnicity or region, so percentages may not represent all records equally.

Navigate back to Table 1.

## Table 2 Long description

The table compares average annual rainfall, average temperature, and altitude between hot spots and cold spots for scabies and pediculosis in Ecuador in 2021, with a reported p value for each comparison. For scabies, hot spots had higher rainfall than cold spots (about 3753 versus 1296 millimetres per year) and higher temperature (about 21.7 versus 12.9 degrees Celsius), and both differences were statistically significant. Scabies hot spots were also at lower altitude than cold spots (about 930 versus 2614 metres above sea level), with a statistically significant difference. For pediculosis, hot spots were warmer than cold spots (about 23.9 versus 0.18 degrees Celsius). Pediculosis hot spots were much lower in altitude than cold spots (about 1033 versus 58.8 metres above sea level), and this altitude difference was statistically significant. The rainfall comparison for pediculosis does not indicate a statistically significant difference based on the reported p value. Interpret results as associations between location type and climate variables, not as evidence of causation.

Navigate back to Table 2.

## Table 3 Long description

The table reports ordinary least squares regression results linking municipal incidence per 1,000 people to temperature in degrees Celsius and annual rainfall in millimeters, modeled separately for scabies and pediculosis. For scabies, both predictors are positive and statistically significant: temperature has a coefficient of 0.115 with standard error 0.035 and p value 0.001, and rainfall has a coefficient of 0.0014 with standard error 0.0002 and p value below 0.001. The scabies intercept is negative and significant, with coefficient minus 2.661, standard error 0.763, and p value below 0.001. Model fit for scabies is moderate, with R squared 0.243 and adjusted R squared 0.236, and the overall model test is significant with F 34.54 and p value below 0.001. For pediculosis, rainfall is positive and significant with coefficient 0.0006, standard error 0.0002, and p value below 0.001, while temperature is not significant with coefficient 0.014, standard error 0.036, and p value 0.643. The pediculosis intercept is not significant, with coefficient 0.265, standard error 0.650, and p value 0.684. Pediculosis model fit is weaker than scabies, with R squared 0.063 and adjusted R squared 0.055, though the overall model test remains significant with F 7.29 and p value 0.001. Coefficients describe associations in incidence per 1,000 for one unit increases in each predictor and do not by themselves establish causation.

Navigate back to Table 3.
