# Supplementary material for: Human scabies and pediculosis in Ecuador: spatial distribution and environmental determinants
Source: Parasitology. 2026 Apr 24;153(6):823–30. doi: 10.1017/S0031182026102078 (PMC13420144; doi:10.1017/S0031182026102078)
Supplement: Vinueza-Veloz et al. supplementary material [file S0031182026102078sup001.docx]

**Table S1.** Municipal incidence of scabies and pediculosis in Ecuador in 2021

| **Municipality** | **Province** | **inc_pediculosis** | **inc_scabies** |
| --- | --- | --- | --- |
| GUARANDA | BOLIVAR | 0.163 | 0.707 |
| CHILLANES | BOLIVAR | 0.000 | 0.862 |
| CHIMBO | BOLIVAR | 0.000 | 0.000 |
| ECHEANDIA | BOLIVAR | 0.000 | 2.724 |
| SAN MIGUEL | BOLIVAR | 0.073 | 0.991 |
| CALUMA | BOLIVAR | 0.000 | 1.447 |
| LAS NAVES | BOLIVAR | 0.000 | 7.058 |
| TULCAN | CARCHI | 0.035 | 0.705 |
| BOLIVAR | CARCHI | 0.000 | 0.000 |
| ESPEJO | CARCHI | 0.000 | 0.673 |
| MIRA | CARCHI | 0.246 | 2.299 |
| MONTUFAR | CARCHI | 0.033 | 0.393 |
| SAN PEDRO DE HUACA | CARCHI | 0.000 | 0.131 |
| LATACUNGA | COTOPAXI | 0.440 | 0.827 |
| LA MANA | COTOPAXI | 0.569 | 1.848 |
| PANGUA | COTOPAXI | 1.639 | 1.867 |
| PUJILI | COTOPAXI | 2.824 | 3.316 |
| SALCEDO | COTOPAXI | 0.103 | 0.825 |
| SAQUISILI | COTOPAXI | 1.066 | 0.750 |
| SIGCHOS | COTOPAXI | 1.048 | 1.868 |
| RIOBAMBA | CHIMBORAZO | 0.146 | 0.390 |
| ALAUSI | CHIMBORAZO | 0.499 | 0.386 |
| COLTA | CHIMBORAZO | 0.245 | 1.090 |
| CHAMBO | CHIMBORAZO | 0.168 | 0.673 |
| CHUNCHI | CHIMBORAZO | 0.788 | 0.079 |
| GUAMOTE | CHIMBORAZO | 1.107 | 0.997 |
| GUANO | CHIMBORAZO | 0.257 | 0.420 |
| PALLATANGA | CHIMBORAZO | 0.087 | 1.213 |
| PENIPE | CHIMBORAZO | 0.445 | 0.890 |
| CUMANDA | CHIMBORAZO | 0.619 | 0.619 |
| ESMERALDAS | ESMERALDAS | 0.058 | 2.992 |
| ELOY ALFARO | ESMERALDAS | 0.025 | 4.781 |
| MUISNE | ESMERALDAS | 0.035 | 6.462 |
| QUININDE | ESMERALDAS | 0.008 | 2.309 |
| SAN LORENZO | ESMERALDAS | 0.047 | 3.225 |
| ATACAMES | ESMERALDAS | 0.048 | 6.285 |
| RIOVERDE | ESMERALDAS | 0.112 | 7.778 |
| IBARRA | IMBABURA | 0.088 | 0.552 |
| ANTONIO ANTE | IMBABURA | 0.092 | 0.368 |
| COTACACHI | IMBABURA | 0.400 | 0.475 |
| OTAVALO | IMBABURA | 0.057 | 0.572 |
| PIMAMPIRO | IMBABURA | 0.308 | 0.154 |
| SAN MIGUEL DE URCUQUI | IMBABURA | 0.191 | 0.893 |
| TENA | NAPO | 1.560 | 11.038 |
| ARCHIDONA | NAPO | 0.481 | 23.069 |
| EL CHACO | NAPO | 0.000 | 3.894 |
| QUIJOS | NAPO | 0.000 | 4.338 |
| CARLOS JULIO AROSEMENA TOLA | NAPO | 0.000 | 4.094 |
| PASTAZA | PASTAZA | 1.709 | 5.805 |
| MERA | PASTAZA | 1.012 | 5.396 |
| SANTA CLARA | PASTAZA | 1.683 | 9.537 |
| ARAJUNO | PASTAZA | 19.257 | 1.849 |
| CAYAMBE | PICHINCHA | 0.128 | 0.641 |
| MEJIA | PICHINCHA | 0.061 | 0.627 |
| PEDRO MONCAYO | PICHINCHA | 0.362 | 0.784 |
| RUMIÑAHUI | PICHINCHA | 0.035 | 0.396 |
| SAN MIGUEL DE LOS BANCOS | PICHINCHA | 0.114 | 2.106 |
| PEDRO VICENTE MALDONADO | PICHINCHA | 0.310 | 2.089 |
| PUERTO QUITO | PICHINCHA | 0.245 | 2.250 |
| AMBATO | TUNGURAHUA | 0.164 | 0.706 |
| BAÑOS DE AGUA SANTA | TUNGURAHUA | 0.050 | 1.149 |
| CEVALLOS | TUNGURAHUA | 0.000 | 1.470 |
| MOCHA | TUNGURAHUA | 0.000 | 0.590 |
| PATATE | TUNGURAHUA | 0.000 | 0.741 |
| QUERO | TUNGURAHUA | 0.000 | 0.989 |
| SAN PEDRO DE PELILEO | TUNGURAHUA | 0.018 | 0.513 |
| SANTIAGO DE PILLARO | TUNGURAHUA | 0.365 | 0.834 |
| TISALEO | TUNGURAHUA | 0.000 | 0.165 |
| LAGO AGRIO | SUCUMBIOS | 0.142 | 4.970 |
| GONZALO PIZARRO | SUCUMBIOS | 0.465 | 6.745 |
| PUTUMAYO | SUCUMBIOS | 0.000 | 7.470 |
| SHUSHUFINDI | SUCUMBIOS | 2.482 | 4.016 |
| SUCUMBIOS | SUCUMBIOS | 0.590 | 0.885 |
| CASCALES | SUCUMBIOS | 0.450 | 6.394 |
| CUYABENO | SUCUMBIOS | 0.280 | 2.243 |
| ORELLANA | ORELLANA | 0.412 | 4.643 |
| AGUARICO | ORELLANA | 10.316 | 27.233 |
| LA JOYA DE LOS SACHAS | ORELLANA | 0.718 | 3.432 |
| LORETO | ORELLANA | 1.654 | 5.340 |
| SANTO DOMINGO | SANTO DOMINGO | 0.342 | 2.247 |
| LAS GOLONDRINAS | ZONA NO DELIMITADA | 0.000 | 0.000 |
| EL PIEDRERO | ZONA NO DELIMITADA | 0.000 | 0.000 |
| QUITO | PICHINCHA | 0.050 | 0.343 |
| LA CONCORDIA | ESMERALDAS | 0.349 | 5.079 |
| CUENCA | AZUAY | 0.016 | 0.216 |
| GIRON | AZUAY | 0.159 | 0.159 |
| GUALACEO | AZUAY | 0.047 | 0.492 |
| PAUTE | AZUAY | 0.039 | 0.039 |
| PUCARA | AZUAY | 0.000 | 0.597 |
| SAN FERNANDO | AZUAY | 0.000 | 0.250 |
| SANTA ISABEL | AZUAY | 0.109 | 0.598 |
| SIGSIG | AZUAY | 0.037 | 0.669 |
| OÑA | AZUAY | 0.000 | 0.837 |
| CHORDELEG | AZUAY | 0.000 | 0.398 |
| EL PAN | AZUAY | 0.000 | 0.000 |
| SEVILLA DE ORO | AZUAY | 0.000 | 0.000 |
| GUACHAPALA | AZUAY | 0.000 | 0.000 |
| CAMILO PONCE ENRIQUEZ | AZUAY | 0.182 | 6.046 |
| AZOGUES | CAÑAR | 0.014 | 0.171 |
| BIBLIAN | CAÑAR | 0.048 | 0.384 |
| CAÑAR | CAÑAR | 0.017 | 0.202 |
| LA TRONCAL | CAÑAR | 0.055 | 1.030 |
| EL TAMBO | CAÑAR | 0.000 | 0.211 |
| DELEG | CAÑAR | 0.000 | 0.328 |
| SUSCAL | CAÑAR | 0.000 | 0.797 |
| MACHALA | EL ORO | 0.065 | 2.439 |
| ATAHUALPA | EL ORO | 0.000 | 0.686 |
| BALSAS | EL ORO | 0.292 | 3.207 |
| CHILLA | EL ORO | 0.000 | 0.403 |
| EL GUABO | EL ORO | 0.020 | 3.099 |
| MARCABELI | EL ORO | 0.000 | 1.651 |
| PASAJE | EL ORO | 0.041 | 1.950 |
| PIÑAS | EL ORO | 0.038 | 1.655 |
| PORTOVELO | EL ORO | 0.082 | 2.623 |
| SANTA ROSA | EL ORO | 0.043 | 3.607 |
| ZARUMA | EL ORO | 0.083 | 0.788 |
| LAS LAJAS | EL ORO | 0.000 | 0.417 |
| GUAYAQUIL | GUAYAS | 0.051 | 0.746 |
| ALFREDO BAQUERIZO MORENO | GUAYAS | 0.040 | 0.596 |
| BALAO | GUAYAS | 0.000 | 1.511 |
| BALZAR | GUAYAS | 0.000 | 0.908 |
| COLIMES | GUAYAS | 0.000 | 0.342 |
| DAULE | GUAYAS | 0.008 | 0.357 |
| DURAN | GUAYAS | 0.021 | 0.496 |
| EMPALME | GUAYAS | 0.027 | 1.652 |
| EL TRIUNFO | GUAYAS | 0.022 | 1.005 |
| MILAGRO | GUAYAS | 0.006 | 0.396 |
| NARANJAL | GUAYAS | 0.029 | 2.434 |
| NARANJITO | GUAYAS | 0.054 | 0.914 |
| PALESTINA | GUAYAS | 0.124 | 0.436 |
| PEDRO CARBO | GUAYAS | 0.069 | 0.967 |
| SAMBORONDON | GUAYAS | 0.015 | 1.465 |
| SANTA LUCIA | GUAYAS | 0.051 | 0.899 |
| SALITRE | GUAYAS | 0.017 | 0.453 |
| SAN JACINTO DE YAGUACHI | GUAYAS | 0.066 | 0.951 |
| PLAYAS | GUAYAS | 0.072 | 1.145 |
| SIMON BOLIVAR | GUAYAS | 0.039 | 0.981 |
| CRNEL. MARCELINO MARIDUEÑA | GUAYAS | 0.000 | 0.166 |
| LOMAS DE SARGENTILLO | GUAYAS | 0.000 | 1.249 |
| NOBOL | GUAYAS | 0.102 | 0.357 |
| GNRAL. ANTONIO ELIZALDE | GUAYAS | 0.000 | 1.034 |
| ISIDRO AYORA | GUAYAS | 0.000 | 0.276 |
| LOJA | LOJA | 0.121 | 0.568 |
| CALVAS | LOJA | 0.142 | 0.674 |
| CATAMAYO | LOJA | 0.033 | 0.359 |
| CELICA | LOJA | 0.069 | 0.415 |
| CHAGUARPAMBA | LOJA | 0.000 | 0.559 |
| ESPINDOLA | LOJA | 0.338 | 0.878 |
| GONZANAMA | LOJA | 0.315 | 0.393 |
| MACARA | LOJA | 0.158 | 0.158 |
| PALTAS | LOJA | 0.210 | 1.260 |
| PUYANGO | LOJA | 0.064 | 0.451 |
| SARAGURO | LOJA | 1.193 | 0.696 |
| SOZORANGA | LOJA | 0.134 | 0.134 |
| ZAPOTILLO | LOJA | 2.843 | 0.650 |
| QUILANGA | LOJA | 0.000 | 0.000 |
| OLMEDO | LOJA | 0.000 | 2.669 |
| BABAHOYO | LOS RIOS | 0.013 | 1.535 |
| BABA | LOS RIOS | 0.000 | 0.832 |
| MONTALVO | LOS RIOS | 0.000 | 0.993 |
| PUEBLOVIEJO | LOS RIOS | 0.000 | 1.535 |
| QUEVEDO | LOS RIOS | 0.017 | 0.997 |
| URDANETA | LOS RIOS | 0.034 | 0.888 |
| VENTANAS | LOS RIOS | 0.000 | 1.217 |
| VINCES | LOS RIOS | 0.028 | 0.822 |
| PALENQUE | LOS RIOS | 0.000 | 0.672 |
| BUENA FE | LOS RIOS | 0.016 | 1.378 |
| VALENCIA | LOS RIOS | 0.023 | 3.313 |
| MOCACHE | LOS RIOS | 0.000 | 1.511 |
| QUINSALOMA | LOS RIOS | 0.000 | 4.431 |
| PORTOVIEJO | MANABI | 0.464 | 3.268 |
| BOLIVAR | MANABI | 0.123 | 2.087 |
| CHONE | MANABI | 0.293 | 1.312 |
| EL CARMEN | MANABI | 0.157 | 2.820 |
| FLAVIO ALFARO | MANABI | 0.040 | 0.920 |
| JIPIJAPA | MANABI | 0.422 | 2.363 |
| JUNIN | MANABI | 0.053 | 1.848 |
| MANTA | MANABI | 0.433 | 2.022 |
| MONTECRISTI | MANABI | 0.498 | 1.935 |
| PAJAN | MANABI | 0.620 | 1.457 |
| PICHINCHA | MANABI | 0.364 | 5.621 |
| ROCAFUERTE | MANABI | 1.255 | 2.779 |
| SANTA ANA | MANABI | 0.084 | 1.604 |
| SUCRE | MANABI | 0.297 | 2.064 |
| TOSAGUA | MANABI | 0.078 | 1.278 |
| 24 DE MAYO | MANABI | 0.069 | 1.907 |
| PEDERNALES | MANABI | 0.453 | 2.576 |
| OLMEDO | MANABI | 0.000 | 1.321 |
| PUERTO LOPEZ | MANABI | 0.929 | 2.005 |
| JAMA | MANABI | 0.559 | 3.999 |
| JARAMIJO | MANABI | 0.649 | 1.731 |
| SAN VICENTE | MANABI | 1.226 | 2.633 |
| MORONA | MORONA SANTIAGO | 3.402 | 5.880 |
| GUALAQUIZA | MORONA SANTIAGO | 10.663 | 6.934 |
| LIMON INDANZA | MORONA SANTIAGO | 2.057 | 2.160 |
| PALORA | MORONA SANTIAGO | 1.298 | 3.749 |
| SANTIAGO | MORONA SANTIAGO | 3.443 | 3.335 |
| SUCUA | MORONA SANTIAGO | 3.494 | 5.677 |
| HUAMBOYA | MORONA SANTIAGO | 6.378 | 6.378 |
| SAN JUAN BOSCO | MORONA SANTIAGO | 22.774 | 11.771 |
| TAISHA | MORONA SANTIAGO | 8.515 | 17.573 |
| LOGROÑO | MORONA SANTIAGO | 7.688 | 5.591 |
| PABLO SEXTO | MORONA SANTIAGO | 9.325 | 8.777 |
| TIWINTZA | MORONA SANTIAGO | 1.573 | 1.144 |
| ZAMORA | ZAMORA CHINCHIPE | 0.549 | 2.666 |
| CHINCHIPE | ZAMORA CHINCHIPE | 3.071 | 0.877 |
| NANGARITZA | ZAMORA CHINCHIPE | 3.272 | 3.079 |
| YACUAMBI | ZAMORA CHINCHIPE | 1.542 | 2.057 |
| YANTZAZA | ZAMORA CHINCHIPE | 2.356 | 3.695 |
| EL PANGUI | ZAMORA CHINCHIPE | 2.785 | 3.017 |
| CENTINELA DEL CONDOR | ZAMORA CHINCHIPE | 0.463 | 4.785 |
| PALANDA | ZAMORA CHINCHIPE | 0.371 | 0.742 |
| PAQUISHA | ZAMORA CHINCHIPE | 1.557 | 10.638 |
| SANTA ELENA | SANTA ELENA | 0.035 | 1.735 |
| SALINAS | SANTA ELENA | 0.146 | 6.087 |
| MANGA DEL CURA | ZONA NO DELIMITADA | 0.000 | 0.000 |
| NABON | AZUAY | 0.252 | 1.133 |
| LA LIBERTAD | SANTA ELENA | 0.063 | 1.595 |
| PINDAL | LOJA | 0.000 | 0.694 |
| ARENILLAS | EL ORO | 0.000 | 0.782 |
| HUAQUILLAS | EL ORO | 0.021 | 0.538 |

inc: incidence

**Reference:**

1. Datos abiertos. Catálogo de Datos Abiertos. Available from: <https://datosabiertos.gob.ec/dataset/?organization=ministerio-de-salud-publica>
